# Effects of Health Guidance on Outpatient and Pharmacy Expenditures: A Disease- and Drug-Specific 3-Year Observational Study Using Propensity-Score Matching

**DOI:** 10.2188/jea.JE20120136

**Published:** 2013-07-05

**Authors:** Etsuji Okamoto

**Affiliations:** National Institute of Public Health, Department of Health and Welfare Service Research, Wako, Saitama, Japan; 国立保健医療科学院

**Keywords:** health guidance, health insurance claims, propensity-score matching, PDM (proportional distribution method), metabolic syndrome

## Abstract

**Background:**

Evidence is lacking on whether health guidance for metabolic syndrome reduces health care expenditures. The author used propensity-score matching to evaluate the effects of health guidance on health care expenditure.

**Methods:**

Men who did and did not receive health guidance from a health insurance society (approximately 60 000 covered lives) were matched (*n* = 397 respectively) using propensity scores. Health insurance claims were compared using cumulative health care expenditures for metabolic syndrome-related outpatient medical care and drug costs for the period from the initial consultation to 3 years later.

**Results:**

No difference was observed between intervention and control groups in cumulative outpatient charges or drug costs related to metabolic syndrome. However, regression analysis using the Tobit model showed that health guidance resulted in a small, nonsignificant reduction in health care expenditure.

**Conclusions:**

Health guidance for metabolic syndrome did not reduce outpatient charges or drug costs related to metabolic syndrome during the 3-year period after the intervention. Findings from Tobit regression suggest that health guidance might eventually result in savings, but this hypothesis remains untested.

## Abstract (ja)

**【背景】:**

メタボリック症候群を対象とする特定保健指導が医療費にどのような影響を及ぼすかについてエビデンスは十分ではない。

**【目的】:**

プロペンシティスコア(傾向得点)マッチングを用いて，特定保健指導の医療費に及ぼす影響を傷病別，薬効分類別に評価する。

**【方法】:**

加入者数約６万人の健康保険組合の男性被保険者のうち2008年度に特定健康診査を受け，メタボリック症候群該当として特定保健指導の対象となった者のうち，特定保健指導を受けた者とそうでない者をプロペンシティスコア(傾向得点)を用いてマッチングし，397組の介入群と対照群について，メタボリック症候群関連の医療費(比例配分法で客観的に推計)とメタボリック症候群を適応症とする薬剤費を初回面接から３年間の累積額で比較した。

**【結果】:**

メタボリック症候群関連の外来医療費ならびに調剤薬剤費に介入群対照群の間に差はなかった。ただ，トビットモデルを用いた回帰分析では，特定保健指導受診群に有意ではないが軽い医療費削減効果が観察された。

**【結論】:**

メタボリック症候群関連の外来医療費と薬剤費については３年間の観察期間では特定保健指導による削減効果はみられなかった。トビット回帰分析では，特定保健指導により将来的には医療費削減効果のある可能性が示唆されたが，その検証は今後に残された課題である。

## INTRODUCTION

As part of the Health Care Expenditure Containment Plans (HCECP), a program of health checks and guidance regarding metabolic syndrome was launched in April 2008. The government estimated that this program would reduce national health care expenditure by approximately 1.6 trillion yen from the expected 49 trillion yen in fiscal year (FY) 2015 and by 2.8 trillion yen from the expected 69 trillion yen in FY 2025 according to data submitted to the cabinet by the Ministry of Health, Labour and Welfare in March 2005.^1^ Pursuant to the Elderly Health Care Security Act, the HCECP at the prefectural level should include forecasts of health care expenditure in each prefecture. However, the national guideline on the HCECP, published in March 2008,^2^ waives the requirement for a health care expenditure forecast during the initial 5-year phase (2008–2012). The guideline states that, “Health checks and guidance will reduce the incidence of metabolic syndrome but will not reduce the number of patients already under treatment. Therefore, it will be some time before the effects of health checks and guidance on health care expenditure become clear. Hence reduction of health care expenditure is expected in the second phase, starting in FY 2013”.

These statements reflect experience of the National Health Insurance (NHI) Health-up Model Projects conducted during 2002–6, which failed to show a reduction in health care expenditure, at least over the short term.^3^ Moreover, there is no established method to evaluate the effects of health checks and guidance on health care expenditure. The health check and guidance program is already under way, and thus it is not possible to adopt an experimental design, like that used for the NHI Health-up Model Projects.

The author used a propensity-score matching technique to develop comparable intervention and control groups. Propensity-score matching is often used for quasi-randomized pharmacoepidemiologic trials that use a large dataset.^4^ The author applied the same methodology to the beneficiaries of a health insurance society, to compare cumulative outpatient charges and drug costs to those of a propensity score-matched control group.

## METHODS

This study was conducted as part of an interim evaluation of the health checks and guidance program of a health insurance society, as established in its executive plan. The author made a contractual agreement with the society, and all personally identifiable data were treated pursuant to the “Guideline for Proper Treatment of Personal Data in Health Insurance Societies”.^5^

The analysis included health checks and guidance data provided in FY 2008 (generally started in September or later) as well as medical and pharmacy claims submitted for treatment rendered in April 2008 thru January 2012. Note that DPC (diagnosis-procedure-combination: the per-diem reimbursement system for acute inpatient care) data were not included, and only claims submitted in electronic form were analyzed (ie, claims in paper form were excluded). Medical claims include both inpatient and outpatient claims, but only outpatient claims were used in the present analysis.

Personal identity was removed from health checks and guidance data and claims data and were replaced with unique numbers linkable through the dataset (details of the procedure are explained elsewhere).^6^ Almost all pharmacy claims were entered into the dataset by April 2008, but only 38.9% of medical claims were entered at that time. The proportion of electronic medical claims has constantly increased, to over 90% in June 2010.

### Modeling

The model was developed by setting health care expenditure (both outpatient medical claims and drug costs) as outcome, health guidance as intervention, and data from health checks and questionnaires as covariates used for propensity scores to predict the probability of receiving health guidance. According to Hoshino, covariates that precede both the outcome and intervention can be regarded as affecting both variables and may thus be used as covariates.^7^ The health check data and questionnaires preceded both health guidance and health care expenditures and could therefore be used as covariates.

**Figure 1.**
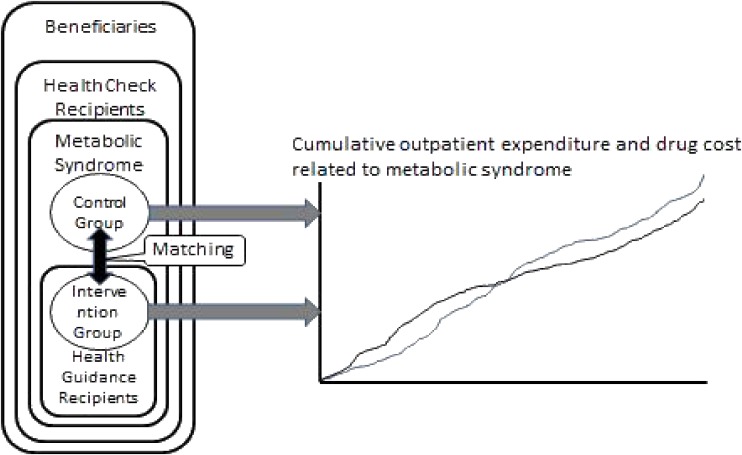
Modeling

The intervention group (men with metabolic syndrome who received health guidance) and the control group (men with metabolic syndrome who did not receive health guidance) were matched 1-to-1 using propensity scores, and cumulative health expenditures were compared (Figure 1).

### Target population

The subjects were male beneficiaries of a health insurance society in the manufacturing industry, with 26 753 insured (88.1% men, average age 40.8 years) and 32 439 dependent family members as of February 2010. Of 59 274 beneficiaries (insured persons and their dependent family members) as of April 2008, a total of 12 754 (9614 men) beneficiaries aged 40 years or older received health checks in FY 2008. Of them, a total of 2572 (2228 men) fulfilled the criteria for metabolic syndrome and were categorized as candidates for health guidance (for men: 1595 for “aggressive” guidance and 633 for “motivational” guidance). Of them, a total of 659 beneficiaries (581 men) received health guidance (for men: 379 received aggressive guidance and 202 received motivational guidance).

### Matching

Beneficiaries who received health checks were classified into 3 categories: aggressive intervention, motivational intervention, and information only. For the first 2 categories, invitations to health guidance were offered by the health insurance society, but recipients were ultimately responsible for whether they received such health guidance. For the purposes of comparison, the control group should consist of beneficiaries who did not receive health guidance but had the same probability to receive such guidance as the intervention group (those who received health guidance).

To predict the probability of a beneficiary receiving health guidance, propensity score (PS)^8^ was calculated using *PSmatch2*, an add-on program of STATA (version 4.0.4, released on 10 Nov 2010).^9^ The logistic regression was done using beneficiaries categorized into the aggressive or motivational interventions, with receipt of health guidance as a target variable (dichotomized to 0, 1) and all laboratory data from health checks (standardized to follow a normal distribution) and questionnaire responses (dichotomized to 0, 1) as covariates.

**Figure 2.**
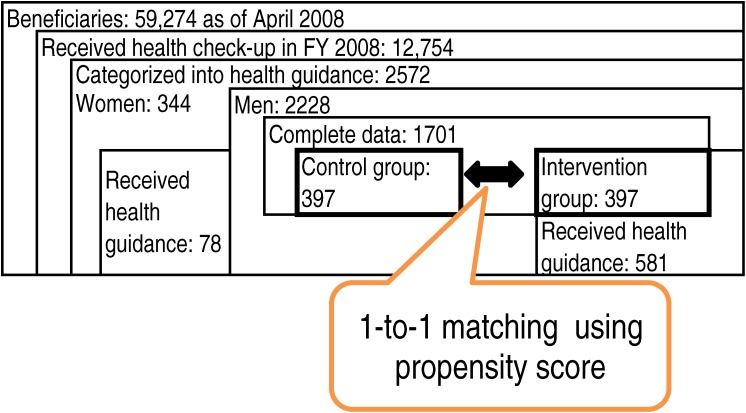
Structure of data

Laboratory data were checked for collinearity, and some data were excluded—ie, body weight, waist size, diastolic blood pressure, aspartate aminotransferase (AST), red blood cell (RBC) count, and hematocrit—because they had high correlation coefficients with BMI (body mass index), systolic blood pressure, alanine amino transferase (ALT), and hemoglobin. Because *PSmatch2* requires a complete dataset with no missing values, a total of 1701 male beneficiaries (397 of whom received health guidance) with complete data were eventually used for matching (Figure 2).

**Figure 3.**
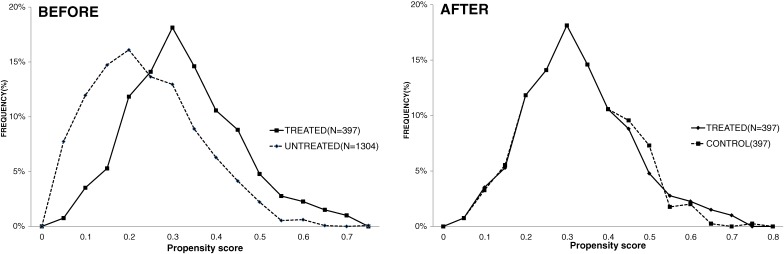
Distribution of propensity score before/after matching

Accuracy was evaluated using the area under the curve (AUC) of the receiver operating characteristics (ROC) curve, and goodness-of-fit (GOF) was evaluated using the Hosmer-Lemeshow test.^10^ Various explanatory variables (covariates) were tested, to achieve best accuracy and goodness-of-fit using STATA. The best accuracy (AUC, 0.7) and goodness-of-fit (Hosmer-Lemeshow value, 0.93) were achieved with the following variables: laboratory data—age, height, BMI, systolic blood pressure, triglyceride, high-density lipoprotein (HDL) cholesterol, ALT, and HbA1c; dichotomous data (1 if any, 0 if otherwise)—past history of any diseases/cerebrovascular diseases/cardiovascular diseases, self-perceived symptoms, glycosuria, proteinuria, stratification of health guidance (1 for aggressive intervention, 0 for motivational intervention), smoking, exercise, walking habit, walking speed, weight change in a year, eating fast, eating at night, eating between meals, skipping breakfast, drinking daily, sleeping well, and desire for health guidance. The distributions of propensity scores before and after matching between those who received health guidance (*n* = 397) and those who did not (*n* = 1304) are shown in Figure 3.

Then, for each beneficiary receiving health guidance, a control was chosen from the group not receiving such guidance. Nearest-neighbor matching was done on a 1-to-1 basis, without replacement.

The control group comprised beneficiaries who were categorized as requiring health guidance but did not receive it. For the purpose of analysis, the beneficiary in the intervention group and his matching control were assumed to have received health guidance on the same date. This assumption allowed control and intervention groups to have the same person-months of observation.

Ultimately, 397 pairs from the intervention and control groups were selected. Balance was checked using standardized difference, and the groups were comparable^11^ (Table 1). A total of 794 beneficiaries from the intervention and control groups were included in the analysis.

**Table 1.** Characteristics of intervention and control groups

Laboratory data	Intervention (*n* = 397)	Control (*n* = 397)	SD for intervention group	Standardized difference
Age (years)	49.0	49.0	6.3	0.00
Height (cm)	171.4	171.1	6.1	0.05
Weight (kg)	76.0	75.5	8.8	0.05^a^
BMI (kg/m^2^)	25.8	25.8	2.5	0.02
Waist circumference (cm)	91.1	90.5	5.9	0.10^a^
Systolic blood pressure (mm Hg)	130.2	129.5	11.8	0.06
Diastolic blood pressure (mm Hg)	82.8	81.7	9.0	0.13^a^
Triglyceride (mg/dl)	153.8	154.3	103.1	0.00
HDL (mg/dl)	54.7	54.2	13.1	0.04
LDL (mg/dl)	139.0	135.7	29.9	0.11
AST (U/l)	25.0	26.0	8.3	0.12
ALT (U/l)	32.9	33.7	18.0	0.04^a^
GTP (U/l)	57.1	69.3	44.0	0.28
HbA1c (%)	5.2	5.2	0.6	0.04
Hct (%)	47.1	46.3	2.9	0.24^a^
RBC (10 000/m^3^)	498.8	495.7	34.3	0.09^a^
Hb (g/dl)	15.5	15.4	1.0	0.07

Questionaire	Intervention (*n* = 397)	Control (*n* = 397)		

Past history of any diseases (none)	140	140		
Self-perceived symptoms (none)	342	344		
Presence of objective symptoms	0	0		
Urine sugar (positive)	13	12		
Urine protein (positive)	13	13		
Use of antihyptertensive drugs	0	0		
Use of antidiabetes drugs	0	0		
Use of antilipidemia drugs	0	0		
Past history of cerebrovascular diseases	2	0		
Past history of cardiovascular diseases	9	6		
Past history of kidney diseases	0	0		
Past history of anemia	0	2		
Smoking	190	187		
Weight change ≥10 kg since age 20 years	270	277		
Regular exercise (≥30 minutes/day >1 year)	63	70		
Regular walking (≥1 hour/day)	53	58		
Walking faster than others of same age	67	71		
Weight change ≥3 kg during previous year	75	75		
Eating faster than others of same age	180	183		
Eating at night (≥3 times/week)	154	146		
Skipping breakfast (≥3 times/week)	51	41		
Drinking alcohol	275	265		
Sleeping and resting well	96	95		
Desire to receive health guidance	203	197		

SD: standard deviation, BMI: body mass index, HDL: high density lipoprotein, LDL: low density lipoprotein, AST: aspartate aminotransferase, ALT: alanine aminotransferase, GTP: glutamyl transpeptidase, HbA1c: hemoglobin A1c, Hct: hematocrit, RBC: red blood cell, Hb: hemoglobin.

^a^Not included in propensity score analysis due to collinearity.

### Health insurance claims

A total of 918 346 outpatient claims and 633 550 pharmacy claims had been submitted electronically to the health insurance society for the 46-month period April 2008 to January 2012. Of them, 4400 outpatient claims and 2646 pharmacy claims were for the intervention group and 3655 outpatient claims and 3138 pharmacy claims were for the control group. The ratio of the number of electronic claims between the 2 groups (intervention/control) was checked to determine if there was bias due to the limited digitization of records in the early phase of observation. The ratios were comparable: 1.28 during the early phase (April 2008 to March 2009) and 1.23 during the late phase (April 2011 to January 2012). Hence the limited digitization of records in the early phase is not likely to have biased the comparison between groups.

Baseline claims data from the control and intervention groups 1 month before the intervention (health guidance) are shown in Table 2. Only 36 persons in the control group and 40 in the intervention group (*n* = 397 for both) had outpatient claims in the month before health guidance. Although the total charges in the intervention group were higher than in the control group (483 970 JPY vs 326 220 JPY), the difference was smaller for metabolic syndrome-related charges (estimated by proportional distribution method [PDM], 51 110 JPY vs 47 740 JPY) and for total charges combining outpatient and pharmacy charges (761 770 JPY vs 737 220 JPY). Hence the intervention and control groups were comparable with regard to baseline charges.

**Table 2.** Baseline claims data from intervention and control groups (*n* = 397 respectively)

Claims before health check (2 months before)								
	*n* of persons		*n* of claims		*n* of visits		Sum of charges	
				
	Control	Intervention	Control	Intervention	Control	Intervention	Control	Intervention
				
Outpatient	17	10	17	10	19	11	9368	10 037
Pharmacy	41	14	47	16	53	19	32 003	12 984
				
Total	58	24	64	26	72	30	41 371	23 021

Claims before health guidance (1 month before)								
	*n* of persons		*n* of claims		*n* of visits		Sum of charges	
				
	Control	Intervention	Control	Intervention	Control	Intervention	Control	Intervention
				
Outpatient	36	40	41	44	56	49	32 622	48 397
(Metabolic syndrome-related charges estimated by PDM)							(4774)	(5111)
Pharmacy	45	39	53	41	69	45	41 100	27 780
				
Total	81	79	94	85	125	94	73 722	76 177

Intervention month of control group was set to the same as that for the individually matched intervention group.

### Estimation of disease-specific and drug-specific costs

To properly evaluate the effects of health guidance on health care charges related to metabolic syndrome, disease-specific outpatient charges and drug-specific costs were evaluated.

The categories for metabolic syndrome-related disease were defined as diabetes (ICD-10: E10–14), other endocrine and nutritional diseases (ICD-10: E15–90), and hypertension (ICD-10: I10–15). Metabolic syndrome-related drug categories were defined as antihypertensives (214), vasodilators (215), antilipidemics (218), other cardiovascular drugs (219), and antidiabetics (396) (the 3-digit numbers in parentheses are drug classifications based on the Japan Standard Merchandise Classification).

Disease-specific outpatient charges were estimated using the PDM program Ver. 4 (available for free at http://resept.com).^12^ PDM is a method for estimating disease-specific charges in a dataset of health insurance claims containing multiple diagnoses. Health insurance claims commonly contain many diagnoses, and it is difficult to determine how much was charged for diabetes in a claim of, say, 4000 JPY containing diagnoses of diabetes and common cold. If the cost of diabetes is known to be 3 times that of the common cold, one could assume that 3000 JPY was charged for diabetes and 1000 JPY was charged for the common cold. PDM applies this simple theory to a large dataset of health insurance claims. Because all diagnoses contained in a claim must be coded, PDM came into common use only recently, after complete digitization of claims.

When health insurance claims were handled in paper form, disease-specific charges were traditionally estimated by classification of principal diagnoses, but the reliability and accuracy of such estimates has been questioned,^13^ and it is increasingly common to use an objective method like PDM to analyze disease-specific charges.^14^ Diagnoses with the modifier 8002 (suspected or rule-out) were excluded from the analysis, except when suspected or rule-out diagnoses were the only diagnoses in a claim.

## RESULTS

### Summary comparison (individual level)

The number of claims/visits/days and charges incurred after initial consultations were compared between the intervention and control groups (Table 3). The inpatient data are presented for reference but were not included in the matched analysis, due to the small sample size. The outpatient charges are inflated for the intervention group, which suggests that health guidance had inflationary effects. However, it should be remembered that the charges are aggregated and include not only metabolic syndrome-related charges and drugs, but also all diseases and drugs.

**Table 3.** Comparison of claims after initial consultation, in intervention and control groups

	Inpatient^a^		Outpatient		Pharmacy	
		
	Control (*n* = 397)	Intervention (*n* = 397)	Control (*n* = 397)	Intervention (*n* = 397)	Control (*n* = 397)	Intervention (*n* = 397)
*n* of patients	21	18	346	345	303	283
*n* of days/visits						
Sum	269	150	4679	5715	3241	2738
Max	72	40	180	195	129	96
Avg^b^	12.8	8.3	13.5	16.6	10.7	9.7
SD	17.1	9.6	17.2	21.8	14.2	13.3
Min	2	1	1	1	1	1
Charges (for all diagnoses and therapeutic classes, not limited to metabolic syndrome)						
Sum	1 112 062	884 575	4 407 987	5 973 622	2 034 665	1 896 383
Max	194 193	260 124	1 023 897	1 289 634	143 914	147 656
Avg^b^	52 955.3	49 143.1	12 739.8	17 314.8	6715.1	6701.0
SD	60 460.0	64 278.8	55 853.4	72 509.9	12 617.9	15 283.0
Min	50	3889	127	338	95	127

^a^DPC claims not included.

^b^Average for sum divided by no. of patients (≠397).

### Disease- and drug-specific comparison (group level)

**Figure 4.**
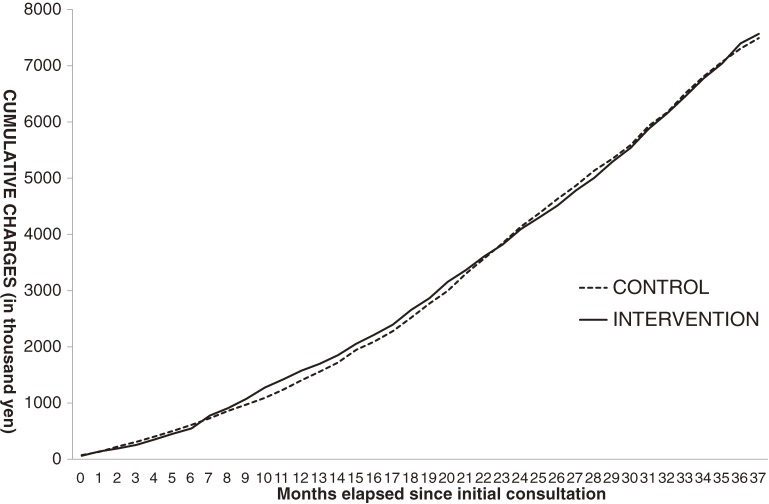
Cumulative charges for metabolic-syndrome-related outpatient care and drug costs of recipients receiving health guidance in FY2008 (intervention group) and propensity-matched controls (*n* = 397, attrition-adjusted)

To evaluate the temporal effects of health guidance, charges were summed from the month when the initial intake consultation was conducted and then compared between the intervention and control groups. Disease-specific outpatient charges were estimated using the PDM, and drug-specific costs were also estimated by drug therapeutic class. Cumulative charges after the initial consultation were compared between the intervention and control groups (Figure 4).

Figure 4 shows the intertwined lines of the intervention and control groups, and suggests that there was no difference in cumulative metabolic syndrome-related outpatient charges or drug costs between the intervention and control groups.

### Tobit regression analysis

In their seminal article Rosenbaum and Rubin proposed 3 techniques to use propensity scores for adjustment: matched sampling, subclassification, and covariance adjustment.^15^ Covariance adjustment is done to conduct a regression analysis using the intervention (receiving health guidance) and propensity scores as explanatory variables. Then the coefficient of intervention variable can be regarded as an intervention effect.

Because health care expenditures are zero-truncated (non-negative) values, and a considerable number of men in both groups had no metabolic syndrome-related outpatient or pharmaceutical charges (94/397 for controls and 114/397 for the intervention group), Tobit regression was conducted using cumulative metabolic syndrome-related outpatient charges and drug costs as a target variable and receipt of health guidance and propensity scores as explanatory variables (Table 4).^16^

**Table 4.** Results of Tobit regression with individual 3-year cumulative metabolic syndrome-related outpatient and pharmaceutical expenditure as target variable

	Coefficient	S.E.	t	*P* > |t|	95% CI	
Received health guidance	−1013.6	1119.2	−0.91	0.365	−3210.7	1183.4
Propensity score	3469.0	4551.6	0.76	0.446	−5465.7	12 403.8
Constant	1438.0	1586.8	0.91	0.365	−1676.8	4552.9
Sigma	15 052.9	451.3			14 167.1	15 938.8

Receipt of health guidance had negative (coefficient = −1013.6) but nonsignificant (*P* = 0.324) effects on metabolic syndrome-related outpatient charges and drug costs.

## DISCUSSION

There is controversy as to whether health checks and guidance will actually reduce health care expenditures. Although public-health professionals believe that prevention will eventually reduce such expenditure, there remains a strong belief that the system will merely waste health care expenditure by increasing unnecessary medication.^17^

Okamura followed a cohort of 4535 National Health Insurance beneficiaries for 10 years and found a positive relationship between per capita health care expenditure and a number of risk factors.^18^ The author claimed that “the finding suggests the possibility of reducing health care expenditure as well as hospitalization and mortality through blood pressure control”. However, the study had limitations: the cohort was not randomized, and no disease-specific health care expenditures were analyzed. A randomized control trial is a better method of evaluating the economic impact of an intervention. Babazono conducted such a trial in an investigation of local residents insured by the National Health Insurance (*n* = 99) and found no significant difference in short-term medical expenses after an intervention.^19^

The present study is strengthened by its use of propensity-score matching for control selection and disease-specific analyses of health care charges. Propensity matching enables quasi-experimentation with observational data if, and only if, there is “strongly ignorable treatment assignment”. Such strong ignorability can be evaluated by calibrating accuracy and goodness-of-fit by means of AUC and the Hosmer-Lemeshow test. This study achieved the best possible control selection, with balance checking. Well-balanced propensity matching can theoretically establish a causal relationship in observational studies.^20^ However, the propensity score methodology has produced controversy and skepticism. One critic, Pearl, claims that “no causal claim can be established by a purely statistical method, be it propensity scores, regression, stratification, or any other distribution-based design”.^21^ Pearl does not deny the theory of propensity scores per se but rather emphasizes the importance of verifying “strong ignorability”. The present author used all possible methods of verification to ensure the validity of propensity-score matching.

Disease-specific analyses of health care charges enable measurement of “pure” health care charges for diseases targeted by interventions. The effects of health guidance for metabolic syndrome related-diseases should be assessed by measuring the health care charges related to metabolic syndrome, not the aggregate charges for unrelated diseases. Objective and reproducible measurement of disease-specific charges was done using PDM instead of the traditional classification by principal diagnoses.^22^ Use of PDM was made possible by digitization of health insurance claims, which include records of all diagnoses. Traditional classification by principal diagnoses is inaccurate and not suitable for economic analyses of this kind.

Use of PDM does not preclude the inherent limitations of diagnoses contained in health insurance claims. Since health insurance claims are not medical documents certified by doctors, their accuracy can always be challenged. The author attempted to minimize this limitation by excluding rule-out diagnoses from the PDM analysis. Rule-out diagnoses account for a considerable portion of diagnoses contained in health insurance claims, and many are recorded to justify reimbursement.^23^ Although diagnoses contained in health insurance claims are not free from bias, PDM analysis that excludes rule-out diagnoses is far more accurate than classification by principal diagnoses.^24^

In this study, a simple comparison of cumulative charges showed no difference between the intervention group and a propensity score-matched control group in the cumulative charges for metabolic syndrome-related outpatient care and drug costs during the 3-year observation period after an initial health guidance consultation. This finding was compatible with the results of an RCT by Babazono^19^ as well as those of a systematic review of “Health-Up” Model Projects by the present author.^3^ However, simple summing of charges cannot distinguish, for example, between frequent but inexpensive users and infrequent but expensive users. A Tobit regression analysis suitable for such zero-truncated data, such as health care expenditures, suggested that health guidance did actually reduce metabolic syndrome-related outpatient charges and drug costs. Although not statistically significant, this finding suggests that health guidance could eventually achieve cost savings over a longer observation period and with a larger cohort. The answer to this is reserved for future research.

This study has some limitations. First, the results are based on male beneficiaries of a health insurance society with approximately 60 000 covered lives and cannot be generalized to women or other insured populations. Similar analyses should be undertaken at different types of insurers, particularly municipal National Health Insurance programs that have more female beneficiaries. Second, the study observation period was limited to the 3 years after the initial consultation. The long-term effects of health guidance on health care charges remain unknown. Continued monitoring will be necessary to evaluate if health guidance achieves cost savings in the long run and, if so, when. Third, the study did not include inpatient claims, due to the possibility that the results could be unduly affected by a small number of expensive hospitalizations. Economic effects, including inpatient claims, should be analyzed in a much larger sample. Last, the study only analyzed electronic claims and did not analyze claims in paper form. Since only 38.9% of medical claims were digitized in the first month of follow-up, it is possible that exclusion of paper claims might have biased the results.

Exclusion of paper claims was inevitable because disease-specific analyses and drug classification can only be determined by using digitized claims. The author found that exclusion of paper claims did not result in substantial bias between intervention and control groups because it was highly unlikely that either group was more inclined to visit clinics that submitted paper claims. By July 2010, the proportion of digitized medical claims reached 90%, at which point any bias would be negligible.

It should be noted that this study only examined effects on health care charges and does not address the medical effectiveness of health guidance, such as improvement of health status. Therefore, the present results should not be interpreted so as to negate the value of health guidance per se.

## ONLINE ONLY MATERIALS

Abstract in Japanese.
