# Supplementary material for: Effects of Health Guidance on Outpatient and Pharmacy Expenditures: A Disease- and Drug-Specific 3-Year Observational Study Using Propensity-Score Matching
Source: J Epidemiol. 2013 Jul 5;23(4):262–9. doi: 10.2188/jea.JE20120136 (PMC3709548; doi:10.2188/jea.JE20120136)
Supplement: Abstract in Japanese. [file je-23-262-s001.pdf]

特定保健指導の外来医療費及び調剤薬剤費に及ぼす影響: 健康保険組合男性被保険者を対象としたプロペンシティスコア(傾向得点)マッチングによる３年間の傷病・薬効別分析結果

岡本悦司
国立保健医療科学院

【背景】メタボリック症候群を対象とする特定保健指導が医療費にどのような影響を及ぼすかについてエビデンスは十分ではない。

【目的】プロペンシティスコア(傾向得点)マッチングを用いて，特定保健指導の医療費に及ぼす影響を傷病別，薬効分類別に評価する。

【方法】加入者数約６万人の健康保険組合の男性被保険者のうち 2008 年度に特定健康診査を受け，メタボリック症候群該当として特定保健指導の対象となった者のうち，特定保健指導を受けた者とそうでない者をプロペンシティスコア(傾向得点)を用いてマッチングし，397 組の介入群と対照群について，メタボリック症候群関連の医療費(比例配分法で客観的に推計)とメタボリック症候群を適応症とする薬剤費を初回面接から３年間の累積額で比較した。

【結果】メタボリック症候群関連の外来医療費ならびに調剤薬剤費に介入群対照群の間に差はなかった。ただ，トビットモデルを用いた回帰分析では，特定保健指導受診群に有意ではないが軽い医療費削減効果が観察された。

【結論】メタボリック症候群関連の外来医療費と薬剤費については３年間の観察期間では特定保健指導による削減効果はみられなかった。トビット回帰分析では，特定保健指導により将来的には医療費削減効果のある可能性が示唆されたが，その検証は今後に残された課題である。

キーワード：特定保健指導，レセプト，プロペンシティマッチング，PDM(比例配分法)，メタボリックシンドローム
